# Supplementary material for: The effects of genital myiasis on the diversity of the vaginal microbiota in female Bactrian camels
Source: BMC Vet Res. 2022 Mar 5;18:87. doi: 10.1186/s12917-022-03189-5 (PMC8897907; doi:10.1186/s12917-022-03189-5)
Supplement: Supplementary file 5 — Additional file 5. [file 12917_2022_3189_MOESM5_ESM.zip › MPL201709200_16s_yy/Treat1/css/js_css/jsPDF-master.zip!/jsPDF-master/docs/specs_utils_compare.js.html]

specs/utils/compare.js - Documentation


## Home

### Classes

- jsPDF

### Global

- addFont
- addMetadata
- addPage
- CapJoinStyles
- circle
- ellipse
- getFontList
- http
- lines
- lstext
- output
- rect
- roundedRect
- save
- setDisplayMode
- setDrawColor
- setFillColor
- setFont
- setFontSize
- setFontStyle
- setLineCap
- setLineJoin
- setLineWidth
- setPage
- setProperties
- setTextColor
- text
- triangle

# specs/utils/compare.js

```
/* global XMLHttpRequest, expect */

function loadBinaryResource (url) {
  const req = new XMLHttpRequest()
  req.open('GET', url, false)
   // XHR binary charset opt by Marcus Granado 2006 [http://mgran.blogspot.com]
  req.overrideMimeType('text\/plain; charset=x-user-defined')
  req.send(null)
  if (req.status !== 200) {
    throw new Error('Unable to load file')
  }
  return req.responseText
}

function sendReference (filename, data) {
  const req = new XMLHttpRequest()
  req.open('POST', `http://localhost:9090/${filename}`, true)
  req.onload = e => {
    console.log(e)
  }
  req.send(data)
}

const resetCreationDate = input =>
  input.replace(
    /\/CreationDate \(D:(.*?)\)/,
    '/CreationDate (D:19871210000000+00\'00\'\)'
  )

/**
 * Find a better way to set this
 * @type {Boolean}
 */
window.comparePdf = (actual, expectedFile, suite) => {
  let pdf
  try {
    pdf = loadBinaryResource(`/base/specs/${suite}/reference/${expectedFile}`)
  } catch (error) {
    sendReference(`/specs/${suite}/reference/${expectedFile}`, resetCreationDate(actual))
    pdf = actual
  }
  const expected = resetCreationDate(pdf).trim()
  actual = resetCreationDate(actual.trim())

  expect(actual).toEqual(expected)
}
```

  

Documentation generated by JSDoc 3.4.2 on Sat Oct 08 2016 21:59:09 GMT+0100 (BST) using the docdash theme.
